# Gamma-tocotrienol treatment increased peroxiredoxin-4 expression in HepG2 liver cancer cell line

**DOI:** 10.1186/s12906-015-0590-y

**Published:** 2015-03-13

**Authors:** Farahani Abdul Rahman Sazli, Zakiah Jubri, Mariati Abdul Rahman, Saiful Anuar Karsani, Abdul Gapor Md Top, Wan Zurinah Wan Ngah

**Affiliations:** Department of Biochemistry, Universiti Kebangsaan Malaysia Medical Centre, Kuala Lumpur, Malaysia; Department of Clinical Oral Biology, Faculty of Dentistry, Universiti Kebangsaan Malaysia, Kuala Lumpur, Malaysia; Institute of Biological Sciences, Faculty of Science, University of Malaya and University of Malaya Centre for Proteomics Research (UMCPR), Kuala Lumpur, Malaysia; Malaysian Palm Oil Board, Bangi, Selangor Malaysia

**Keywords:** Proteomics, Gamma-tocotrienol, HepG2 cells, Peroxiredoxin-4

## Abstract

**Background:**

To determine the antiproliferative effect of gamma-tocotrienol (GTT) treatment on differential protein expression in HepG2 cells.

**Methods:**

HepG2 cells were treated with 70 μM GTT for 48 hours and differentially expressed protein spots were determined by two-dimensional electrophoresis (2DE), identified by MALDI-TOF mass spectrometer (MS) and validated by quantitative real-time polymerase chain reaction (qRT-PCR).

**Results:**

GTT treatment on HepG2 cells showed a total of five differentially expressed proteins when compared to their respective untreated cells where three proteins were down-regulated and two proteins were up-regulated. One of these upregulated proteins was identified as peroxiredoxin-4 (Prx4). Validation by qRT-PCR however showed decreased expression of Prx4 mRNA in HepG2 cells following GTT treatment.

**Conclusions:**

GTT might directly influence the expression dynamics of peroxiredoxin-4 to control proliferation in liver cancer.

## Background

Gamma-tocotrienol (GTT) is a member of the vitamin E family and has been reported to have antiproliferative effect against a wide variety of tumor cells such as breast cancer cells [1], human leukemia cells [2], mammary malignant epithelial cells [3], human cervical carcinoma cells [4] and liver cancer cells [5]. Several possible mechanisms have been proposed to explain the antiproliferative activity of GTT either through its antioxidant effect [6,7], enzyme suppression [8,9], pro-apoptotic effects [10,11], mitogenesis regulation [2] or anti-angiogenic activity [12]. In breast cancer cells GTT inhibited the nuclear factor kappa beta (NF-κB) activation pathway, leading to the down-regulation of various NF-κB-downstream targets that support cell survival (IAP1, IAP2, Bcl-xL, Bcl-2, cFLIP, XIAP, Bfl-1/A1, TRAF1, and survivin) and proliferation (cyclin D1, COX2, and c-Myc) besides potentiating apoptosis [13]. The involvement of multiple signaling pathways (NF-κB and JNK) and proteins (EGF-R, Id-1 and Id-3) was suggested to explain the antiproliferative effect of GTT in prostate cancer (PCa) cells [14].

Tocotrienols especially δ- and γ-tocotrienol (GTT) modulates cellular redox status by regulating the expression levels of antioxidant enzymes. GTT increases the expression of several antioxidant enzymes such as thioredoxin and quinine reductase 2 (NQO2) while suppressing the growth of MCF-7 cells [15]. In MDA-MB-231 cells, treatment by tocotrienols led to several fold increase of NRF2 expression marked by corresponding decrease in Kelch-like ECH-associated protein 1 (KEAP1) levels [15]. Major intracellular antioxidants such as glutathione, superoxide dismutase and catalase have long been recognized as part of our body’s detoxification mechanisms. However, a recently identified novel antioxidant from a family of peroxidases, the peroxiredoxins (Prx), has been shown to reduce hydroperoxides with the use of electrons provided by a thioredoxin or a glutaredoxin [16].

Prx exists as six unique isoforms (PrxI to VI) in mammalian cells. They exhibit different expression patterns during development, distribute differently in organelles, and undergo different reaction intermediates during catalysis [17]. Some of these isoforms provide defence against oxidative damage and others appear to participate in signalling by controlling H_2_O_2_ concentration [17]. A connection between Prx and vitamin has recently been established by Tolle et al. [18]. They reported that the expression of Prx1 and Prx6 is selectively regulated in alveolar type II cells isolated from vitamin E-depleted rats, depending on the severity of oxidative load. Another study by Dahlan et al. [19] suggested that tocotrienol rich fraction directly influenced the expression dynamics of peroxiredoxin-2 in H_2_O_2_ challenged lymphocyte cells to improve the cells ability to resist oxidative damage. This suggested that peroxiredoxins are regulated by vitamin E in coping with oxidative stress. The antiproliferative effect of GTT in rat hepatoma dRLh-84 cells and human hepatoma Hep3B cells were reported by Sakai et al. [20,21], in murine liver cancer cells, BNL 1ME A.7R.1 by Har and Keong [22], and in HepG2 cells by Aida et al. [5]. Our earlier study on the HepG2 cell line showed that GTT increased Ras protein expression [5]. Sakai et al. [20,21] and Har and Keong [22], demonstrated a rise in caspases activity during apoptosis induction in several liver cancer cell lines being treated with GTT.

Thus, with that in mind this study we aim to determine the changes in protein expression involved in the antiproliferative activity of GTT in HepG2 cells.

## Methods

### Cell culture and GTT treatment

Hepatoma HepG2 cell lines from the American Type Culture Collection (ATCC, USA) was maintained in Eagle minimum essential medium (EMEM, Flowlab, Australia) containing 10% fetal calf serum (PAA, Austria), penicillin/streptomycin (100 μg/ml) (Flowlab, Australia) at 37°C in an atmosphere containing 5% CO_2_. On reaching confluence, cells were trypsinized, centrifuged and counted using a haemocytometer. Cells were then plated at a consistent density of 2 × 10^6^ cells/100-mm culture plates for every treatment group.

Gamma tocotrienol was supplied by Malaysian Palm Oil Board (MPOB). Stock solution of GTT (0.5 M) was prepared in 100% ethanol and stored as small aliquots at −20°C. Prior to use, GTT from the stock solution was mixed with fetal calf serum and incubated overnight at 37°C. The GTT was then diluted to a 70 μM solution in a mixture of culture medium and 100% ethanol where the final ethanol concentration was less than 0.05%. Cells with the density and condition as stated above were left untreated or treated with GTT at 70 μM final concentration for 48 hours. Untreated cells were cultured only in EMEM without GTT. All treatments were done in triplicate and the experiments were repeated thrice.

### Sample preparation for 2DE

After 48 hours of GTT treatment, cells were harvested by trypsinization and transferred into 15 ml falcon centrifuge tubes. The cells were harvested by centrifugation at 800 rpm for 10 minutes. The resulting pellet was then washed thrice with cold phosphate buffered saline (PBS). It was then re-suspended in 200 μl lysis buffer (8 M urea, 2% CHAPS, 0.5% pH 4–7 IPG buffer, Amersham, USA) containing protease inhibitor mix (Amersham, USA). The cells were then incubated on ice for 30 minutes with intermittent vortexing at 10 minute intervals. After centrifugation at 13000 rpm for 30 minutes at 4°C, the supernatant was transferred to sterile microcentrifuge tubes. Protein concentration was determined by Bradford assay (1976) [23].

### Two-dimensional gel electrophoresis (2DE)

First dimension separation was carried out on immobilized pH gradient (IPG) strips (24 cm, linear, pH 4–7, GE Healthcare Bio-Sciences, Uppsala, Sweden) with Ettan IPGphor II Isoelectric Focusing System and standard strip holder (GE Healthcare Bio-Sciences, Uppsala, Sweden). IPG strips pH 4–7 were used for the 2DE investigation because our preliminary study using pH 3–10 strips indicated that the majority of most affected proteins were found within this region (data not shown). The use of 24 cm pH 4–7 IPG strips improved gel resolution within this region compared to pH 3–10 strips. Protein samples (80 μg) were loaded into sample cups at the anode end. The IPG strips were then focused for a total of 65 kVh. Upon completion of IEF, strips were equilibrated in equilibration buffer (6 M urea, 75 mM Tris–HCl, pH 8.8, 29.3% glycerol, 2% sodium dodecyl sulphate (SDS), 0.002% bromophenol blue, 1% dithiotreitol (DTT)) for 15 minutes, followed by the same buffer containing 25% iodoacetamide instead of DTT for another 15 minutes.

The second dimension separation was carried out at 15°C on 12.5% SDS slab gels using an ETTAN DALT II electrophoresis system (GE Healthcare Bio-Sciences, Uppsala, Sweden), with the IPG strips sealed on the top of the gels with 0.5% agarose. Sodium dodecyl sulphate polyacrylamide gel electrophoresis (SDS-PAGE) was run at constant power (1 W/gel) for 1 hour, then switched to 13 W/gel until the bromophenol blue marker reached the bottom of the gel. For every treatment group, triplicate runs were performed.

### Gel staining and image analysis

Protein spots were visualized by silver staining as described in the plus one silver staining kit (GE Healthcare Bio-Sciences, Uppsala, Sweden). The complete protocol was applied for analytical gels. For preparative gels, the protocol was modified so that glutaraldehyde was omitted from the sensitization step and formaldehyde omitted from the silver reaction step. Images of stained 2DE-gels were acquired with a UMAX scanner, model UTA-2100 XL and stored as tagged image file format (TIFF) images. The 2DE maps were then analyzed using the 2D Image Master Platinum software Version 6.0 (GE Healthcare Bio-Sciences, Uppsala, Sweden). Only those spots that changed consistently by more than 2.0 fold were selected as spots of interest.

### In-gel tryptic digestion

Protein spots were excised and in-gel digested using trypsin (Promega) for mass spectrometric analysis. Briefly, excised spots were first destained in destaining solution (15 mM potassium ferricyanide/50 mM sodium thiosulphate, 1:1 [v/v]). The spots were then reduced in a solution containing 10 mM DTT/100 mM ammonium bicarbonate for 30 minutes at 60°C and alkylated in 55 mM iodoacetamide/100 mM ammonium bicarbonate for 20 minutes in the dark. The gel pieces were then washed (3 × 20 minutes) in 50% acetonitrile/100 mM ammonium bicarbonate. This was followed by dehydration of the gel pieces in 100% acetonitrile and drying in a vacuum centrifuge (SpeedVac, Thermo Scientific, Savant DNA 120). Subsequently the dried gel pieces were rehydrated with 25 μl of 7 ng/μl trypsin (Promega trypsin gold) in 50 mM ammonium bicarbonate buffer and digested at 37°C for 18–20 h. Tryptic peptides were then extracted using 50% acetonitrile for 15 minutes, followed by 100% acetonitrile for 15 minutes. The extracted solutions were then pooled into a single tube and dried in a SpeedVac concentrator and solubilized with 10 μl of 10% acetonitrile/40 mM ammonium bicarbonate.

### MALDI-TOF/TOF mass spectrometer analysis and database searching

Extracted proteins were first desalted using ziptip C18 (Millipore, USA) according to protocols described by the manufacturer. The final elution volume following ziptip clean up was 1.5 μl. The peptide samples were then mixed (1:1) with a matrix consisting of a saturated solution of α-cyano-4-hydroxycinnamic acid (CHCA, Sigma) prepared in 50% acetonitrile/0.1% trifluoroacetic acid. Aliquots of samples (0.7 μl) were spotted onto stainless-steel sample target plates. Peptide mass spectra were obtained on a MALDI-TOF/TOF mass spectrometer (ABI 4800 plus, Applied Biosystems) in the positive ion reflector mode. For precursor ion selection, all fractions were measured in a single MS before MS/MS was performed. For MS/MS spectra, the peaks were calibrated by default. The 20 most abundant precursor ions per sample were selected for subsequent fragmentation by high-energy collision-induced dissociation (CID). The collision energy was set to 1 keV and the air was used as the collision gas. The criterion for precursor selection was a minimum S/N of 5. The mass accuracy was within 50 ppm for the mass measurement and 0.1 Da for CID experiments. The other parameters for searching were of trypsin, 1 missed cleavage, variable modification of carbamidomethyl and oxidation of methionine, peptide charge of 1+, and monoisotopic.

For database searches, known contamination peaks such as keratin and auto proteolysis peaks for trypsin were removed before searching. Spectra was processed and analyzed by the Global Protein Server Explorer 3.6 software (Applied Biosystems). This uses an internal MASCOT (Matrix Science, UK) program for matching MS and MS/MS data against database information. The data obtained were screened against human databases downloaded from the Swiss-Prot/TrEMBL homepage (http://www.expasy.ch/sprot).

### Quantitative real time RT-PCR analysis

Total ribonucleic acid (RNA) was extracted from HepG2 cells using TRIzol reagent according to the manufacturer’s instruction (Invitrogen, Paisley, United Kingdom). The total RNA acted as template and was analyzed using script one-step RT-PCR kit with SYBR Green (Biorad, USA) according to the manufacturer’s protocols. Primers were designed with Primer 3 software and blasted with NCBI database sequences. The primers used are shown in Table 1*.*

**Table 1:** **Primers used in quantitative RT-PCR analysis of proteins identified by 2DE**

**Protein**	**Accession number**	**Primer sequence 5′–3′**
PRDX4	NM_006406.1	F: ccacttctacgcgggtggacaa
		R: cagtagggcgctggcttggaaa

PCR amplification of identified gene, Prx4 was performed by iQ5 Multicolor Real Time PCR System (Biorad, USA) using the following program: cDNA synthesis at 50°C for 20 minutes, iScript reverse transcriptase inactivation at 95°C for 4 minutes followed by 38 amplification cycles of denaturation melting at 95°C for 10 sec and 61°C (primer annealing and extension) for 30 sec. After the last cycle, a melting curve analysis was generated at 95°C for 1 minute, 55°C for 1 minute and 60°C for 10 sec (70 cycles, increase set point temperature after cycle 2 by 0.5°C). The expression levels of targeted genes were normalized against β-actin. The PCR specificity was examined in 1.8% agarose gel.

Quantitative real time PCR was performed to assess the mRNA expression of Prx4 proteins. This performed with samples from HepG2 cells with and without 48 hours of GTT treatment (70 μM).

### Statistical analyses

**Figure 1:**
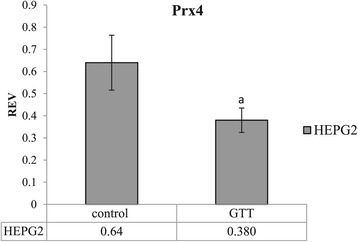
**mRNA expressions of Prx4 in HepG2 cells.** Results represent the mean ± S.D. for three experiments. a indicates a significant decrease of mRNA expression (p < 0.05) in GTT-treated HepG2 cells compared to control untreated HepG2 cells.

Data for mRNA expression of Prx4 in Figure 1 was expressed as mean ± S.D and differences between group was statistically analyzed by t-test for independent samples parametric data distribution and considered significant when p < 0.05. Data analysis was performed using SPSS for Windows, version 17.

## Results

### Differential analysis of 2DE protein maps of HepG2 cells

**Figure 2:**
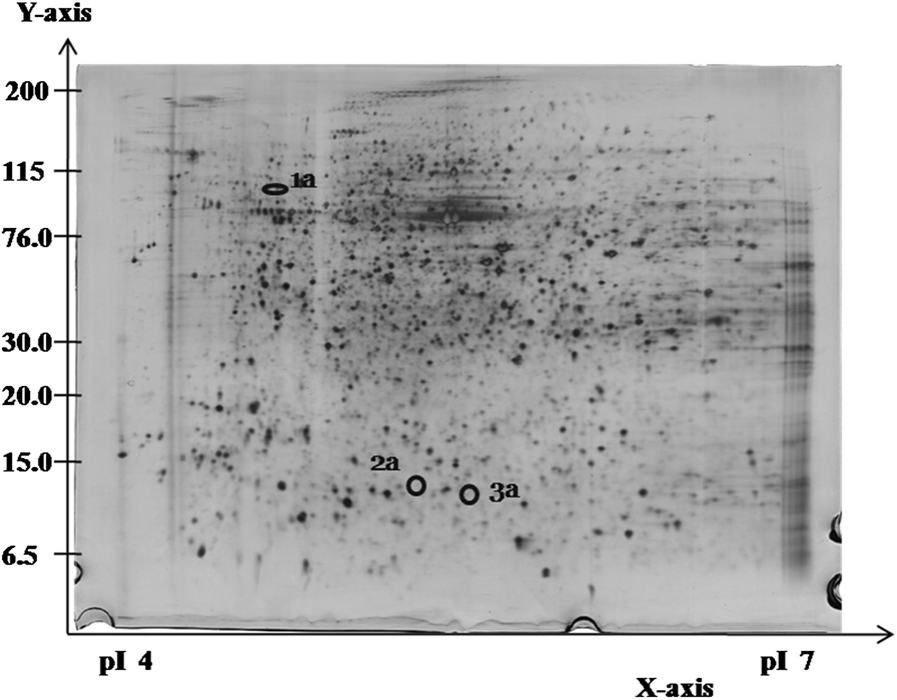
**Representative silver-stained 2DE gel for HepG2 cells without GTT treatment at pH 4–7.** The circled and numbered proteins (1a, 2a and 3a) are the differentially expressed proteins. All these three proteins were down-regulated during GTT treatment and could not be detected in the protein profile of HepG2 cells with GTT treatment.

**Figure 3:**
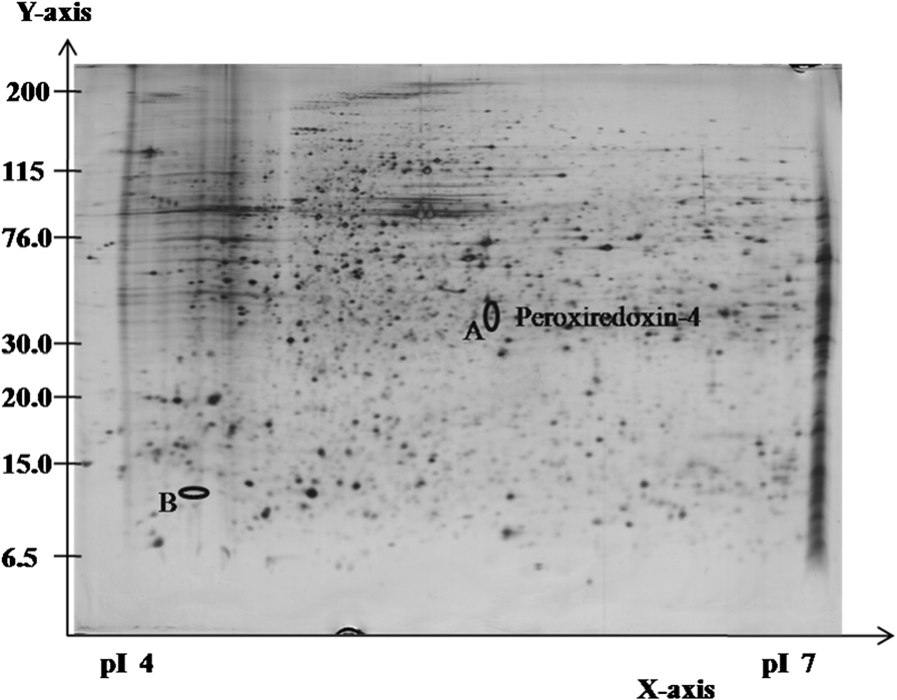
**Representative silver-stained 2DE gel for HepG2 cells with GTT treatment at pH 4–7.** The circled and labelled proteins (A and B) are the differentially expressed proteins. Both proteins were exclusively up-regulated during GTT treatment and could not be detected in the protein profile of control HepG2 cells (without GTT treatment).

**Figure 4:**
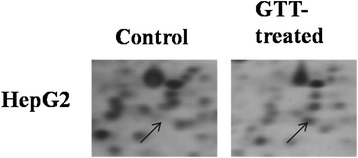
**Enlargement of Prx4 (spot A) without (control) and with GTT treatment of HepG2 cells in 2DE gel.**

The expression patterns of protein spots from HepG2 cells treated with GTT were compared to untreated control using 2DE image analysis software. Differential protein expression data were obtained from an independent comparison of at least three pairs of gels obtained from three independent cell growths. The analysis showed that five protein spots were differentially expressed (≥2.0-fold difference) in HepG2 cell lines compared to untreated control groups. Three protein spots were down-regulated (Figure 2) while two protein spots were up-regulated (Figure 3) with GTT treatment. Out of five protein spots (Table 2), MALDI-TOF/TOF was only able to identify one protein spot which is protein A, peroxiredoxin 4 (Table 3) that is shown in an enlarged diagram in Figure 4.

**Table 2:** **Expression pattern for the differentially expressed proteins in HepG2 cells**

**Protein no.**	**Control untreated (HepG2 cells)**	**GTT-treated HepG2 cells**
1a	+ (∞)	
2a	+ (∞)	
3a	+ (∞)	
A (Prx4)		+ (∞)
B		+ (∞)

+ indicates significant up-regulation of protein expression. ∞ indicates exclusively expressed protein in either control untreated cells or GTT-treated cells.

**Table 3:** **Peroxiredoxin-4 identification by mass spectrometry data**

**No**	**Protein name**	**Abbreviation**	**Swiss-Prot accession no.**	**pI/MW (Experimental)**	**pI/MW (Theoretical)**	**Protein score**	**Peptides (%cov)**
A	Peroxiredoxin 4	Prx 4	Q13162	5.7/33500	5.86/30521	281	6 (36)

### Analysis of prx4 transcript level by qRT-PCR

Quantitative RT-PCR showed that the resulting relative gene expression profiles were not similar to the protein expression profiles observed in 2DE. On the contrary, for HepG2 cells the relative expression observed was the reverse of the protein expression observed in 2DE (Figure 1).

## Discussion

Profile changes in the cellular proteome induced by GTT was observed on whole cell lysates of control and treated HepG2 cells. In the present study, the pH 4–7 IPG strips were chosen for 2DE investigation as most of the affected proteins were found in this region in a preliminary study using pH 3–10 linear IPG strips (data not shown). The usage of 24 cm pH 4–7 IPG strips improve the gel resolution in this region.

2DE successfully resolved approximately 1500 protein spots in all 2DE gels of control and treated HepG2 and WRL-68 cells. Among these proteins, 15 protein spots were either up- or down-regulated by more than 2-fold in HepG2 treated samples versus HepG2 control samples. Out of five protein spots which showed significant expression changes, only one protein spot was able to be identified by MS. This was probably due to low protein quantity making it difficult to obtain good MS spectrums for protein identification. This may be in part due to the use of silver staining in visualizing the protein spots on 2DE gels. Silver staining is one of the best methods for staining 2DE gels because it offers maximal sensitivity despite being a low cost and easy to handle procedure. However, silver staining has been reported to have a rather poor compatibility with MS [24,25]. Modifications of proteins by silver staining may result in low sequence coverage. Omission of glutaraldehyde increased sequence coverage, but this improved sequence coverage especially for low concentration of protein is still not sufficient for secure identification [25]. However, silver staining was still in this study as it provided the best balance between sensitivity of protein detection and protein identification.

A previous study by Aida et al. [5] used the same treatment condition (48 hr GTT treatment) with HepG2 cells but with a higher GTT dose (170 μM). This resulted in increased Ras protein expression. The present study however, used a lower GTT dose (70 μM) to ensure maximum cell viability for the identification of direct molecular targets of GTT. Prx4 was the only protein that was identified by MS and was highly expressed in HepG2 treated cells. This was a novel observation as Prx4 has never before been reported in any GTT and vitamin E anticancer mechanism in general.

Prx4 is ubiquitously expressed and localized in the endoplasmic reticulum (ER) and extracellular space [26,27], with highest expression in the pancreas, liver and heart, and lowest expression in blood leukocytes and the brain [26,28]. It is overexpressed in lung, pancreatic and prostate cancer and has been suggested as a biomarker candidate for all three cancers [29-31]. In bladder and triple-negative breast (a subtype of breast cancer with very poor prognosis) cancer [32], Prx4 was associated with a poor survival and its selective inhibition may serve as additional option for treatment of bladder cancer [33].

The up-regulation of Prx has been reported in various studies in which oxidative stress was involved [34-36]. This up-regulation is a normal cellular response to reduce the resulting oxidative stress, thus implying the occurrence of oxidative stress of GTT treatment in the present study. Kannappan et al. [37] has reported the induction of ROS generation in colon cancer cells, HCT-116, within 10 minutes of GTT treatment. These findings further support the idea of the involvement of oxidative stress in the effects of GTT treatment. The up-regulation of Prx4 in this study may be a response to reduce the threatening effects of increase in ROS or H_2_O_2_ induced by GTT. The observed antiproliferative effect of GTT may be due the high amount of H_2_O_2_ induced by GTT treatment in HepG2 cell. This is supported by a separate study in our lab using N-acetyl cycteine (NAC) as the inhibitor for ROS to confirmed that the action of GTT was through the induction of H_2_O_2_ (unpublished data).

Other studies have also shown that GTT is able to suppress NF-κB activation resulting in proliferation suppression and apoptosis induction in cancer cells [38,39]. The transcription factor NF-κB regulates a number of genes that control cell proliferation and cell survival. In tumor cells, NF-κB is constitutively active resulting in the continuous expression of genes that keep the cells proliferating and protecting them from apoptosis. Prx4 has been suggested as an immediate regulator of H_2_O_2_-mediated activation of NF-κB [28]. In addition, it is also well-established that reactive oxygen intermediates such as H_2_O_2_ can mediate NF-κB activation. NF-κB activation usually ensures cell proliferation and cell survival. Prx4 may prevent H_2_O_2_-induced activation of NF-κB by reducing H_2_O_2_ [28,40]. By relating the antioxidant activity of Prx4 and the ability of NF-κB suppression by GTT, it is possible that Prx4 might indirectly help in suppressing NF-κB activation through H_2_O_2_ metabolism. This may explain the suppression of proliferation in GTT-treated HepG2 cells observed in this study. Future functional studies on the functional role of Prx4 may provide insights in determining the relationship between Prx4 and NF-κB in HepG2 cells.

Quantitative real-time PCR was performed to assess the mRNA expression of Prx4. The qRT-PCR result showed that the mRNA expression of Prx4 did not correspond with its protein abundance as observed in 2DE. This suggested that Prx4 was not regulated at the mRNA level. In contrast to the increased expression of Prx4 protein in HepG2 cells after GTT treatment, Prx4 messenger ribonucleic acid (mRNA) was apparently down-regulated. It has been repeatedly demonstrated that mRNA levels do not always correlate with protein expression levels such as that observed in human liver tissue [41] and capsaicin-treated HepG2 cells [42]. Both studies emphasized the probabilities of the involvement of posttranscriptional or posttranslational modifications and also unknown regulatory mechanisms and signalling. An example of complex regulatory mechanisms in the expression of drosophila peroxiredoxin I (dPrx I) where the existence of two alternative 5′UTRs in the mRNA transcript of dPrx I lead to identical coding sequence: namely Ia and Ib [43]. Ia translation is enhanced in steady-state cells while Ib translation is increased in cells under oxidative stress. If this kind of complexity is regulated by the DNA of a mere insect, higher organism including humans is expected to have a more elaborate and complex cellular and DNA regulatory mechanisms that are still unknown. However, with presently available data, it is not possible to postulate a definite explanation.

## Conclusions

In conclusion, our data suggested that the antiproliferative effect of GTT on HepG2 cells may involve the change in expression of at least five proteins (up-regulation of two proteins and down-regulation of three proteins). We were able to identify one of these proteins as Prx4. However, the exact manner in which the protein function triggering or inducing the suppression of proliferation HepG2 cells remains to be elucidated.
